# Fabric–elasticity relationships of femoral head trabecular bone are similar in Type 2 diabetes and non-diabetic individuals

**DOI:** 10.1016/j.bonr.2025.101884

**Published:** 2025-10-29

**Authors:** Mathieu Simon, Sasidhar Uppuganti, Jeffry S. Nyman, Philippe Zysset

**Affiliations:** a ARTORG Centre for Biomedical Engineering Research, University of Bern, Bern, Switzerland; b Department of Orthopaedic Surgery, Vanderbilt University Medical Center, Nashville, TN 37232, USA; c United States Department of Veterans Affairs, Tennessee Valley Healthcare System, Nashville, TN 37212, USA

**Keywords:** Bone, Diabetes, HR-pQCT, Fabric, Elasticity

## Abstract

Type 2 diabetes (T2D) is a chronic disease leading to an elevated glucose level in the blood and increased fracture risk. Because T2D individuals tend to have normal to higher areal bone mineral density (aBMD) than healthy individuals, their fracture risk is often underestimated. As an alternative, high-resolution peripheral quantitative computed tomography (HR-pQCT) is an attractive tool to investigate bone morphology in vivo and estimate fracture risk. Based on HR-pQCT scans, bone strength can be estimated using micro finite element (μFE) analysis or homogenized finite element (hFE) analysis. While μFE is computationally expensive, hFE provides an accurate estimation of bone mechanical properties within reasonable efforts. However, the hFE scheme is based on relationships between the local fabric (anisotropy) and elasticity. These relationships have been shown to hold for healthy controls as well as in the case of osteogenesis imperfecta. Nevertheless, whether these relationships are also valid for T2D-diagnosed patients remains unclear. Therefore, the present work aims to compare fabric–elasticity relationships between T2D and non-diabetic controls.

The present study collected 56 trabecular bone cores from the femoral head of 28 T2D and 28 control donors. These cadaveric samples were scanned in a micro-CT system at an isotropic 14.8 μm voxel size. Three cubic regions of interest (ROIs) were selected in each scan. The resolution of these ROIs was coarsened by a factor of 4, mimicking clinical HR-pQCT resolution, and the ROIs were subsequently segmented. Standard morphometric parameters were computed from the segmented ROIs using medtool (v4.8; Dr. Pahr Ingenieurs e.U., Pfaffstätten, Austria). Additionally, their fabric tensor and their apparent stiffness tensors were computed. The ROIs were compared between T2D and control regarding their morphometric and mechanical properties. Finally, ROIs were matched between T2D and control for bone volume fraction (ρ) and degree of anisotropy (DA). The matched dataset allowed the comparison of fabric–elasticity relationships between T2D and control samples.

No significant difference was observed between T2D and control samples, both regarding their morphology and their mechanical properties. Specifically, fabric–elasticity relationships were shown to hold for both the control and the T2D groups. A comparison of the resulting exponents related to ρ and DA has highlighted different trends but no important difference between T2D and control samples.

In conclusion, trabecular bone architecture was similar between T2D and non-T2D donors. Additionally, fabric–elasticity relationships, i.e. morphology-mechanical relationships, are also similar between donors with and without diabetes. Accordingly, HR-pQCT-based hFE analysis could also be used for estimating the bone mechanical properties of T2D patients and for their fracture risk assessment.

## Highlights

• Bone structure is similar in T2D and non-diabetic femoral head sample.
• Morphology-mechanics relations are similar between T2D and controls.
• HR-pQCT-based hFE is valid for assessing T2D bone mechanical properties.

## Graphical abstract

**Figure:**
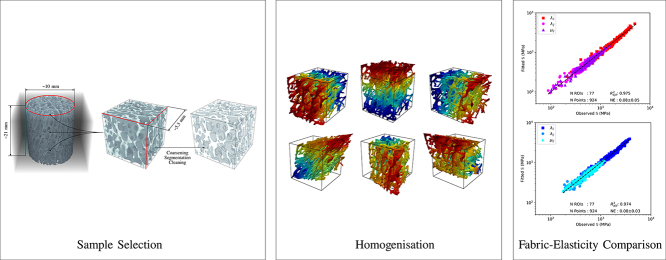

## 1 Introduction

Fragility fractures are, by definition, bones that break under a load which should not have caused a fracture in healthy conditions (Kanis et al., 2001). Such fractures are a worldwide burden, causing high morbidity, mortality, and high costs for the health care system (Burge et al., 2007, Kanis et al., 2021). The lifetime risk of fragility fracture is relatively high, lying between 40%–50% for women and 13%–22% for men (Johnell and Kanis, 2005). This fracture risk is even higher in adults diagnosed with type 2 diabetes (T2D) (Schwartz et al., 2001, Ahmed et al., 2005).

T2D is a chronic disease where cells become insulin resistant, leading to a lower glucose uptake by the cell and thus, an elevated glucose level in the blood (Roglić, 2016). T2D conditions also lead to an increase of advanced glycation endproducts (AGEs) in bone collagen (Makita et al., 1991). AGEs are shown to interfere with reducing bone formation (Adami, 2009) and bone resorption (Valcourt et al., 2007) leading to a reduced bone turnover. Additionally, AGEs form non-enzymatic cross-links across and within collagen fibers, leading to more brittle bone matrix and decreasing toughness (Tang et al., 2009). Thus, T2D can lead an increased bone brittleness and potentially also reduced quality (Lecka-Czernik and Fowlkes, 2015, Rubin and Patsch, 2016).

Currently, fracture risk is usually assessed based on areal bone mineral density (aBMD) from dual-energy X-ray absorptiometry (DXA) measurement performed either at the lumbar spine or the femoral neck (Lespessailles et al., 2017, Nuti et al., 2018). However, T2D patients tend to have normal to higher aBMD than healthy individuals (Ma et al., 2012). This trend might arise from the fact that T2D patients tend to present a higher body mass index, thus, triggering an osteogenic response. Therefore, T2D-associated skeletal fragility is underestimated in clinics (Samelson et al., 2018).

An alternative to DXA is high-resolution peripheral quantitative computed tomography (HR-pQCT). Indeed, as opposed to DXA, which provides areal size-dependent measurement, HR-pQCT provides 3-dimensional size-independent quantitative measurement (Whittier et al., 2020). The high resolution provided by HR-pQCT allows for the analysis of the trabecular and cortical bone phases separately. Additionally, HR-pQCT images can be used as a basis to perform finite element analysis (FEA), allowing the estimation of local bone mechanical properties, which can, in turn, be used for fracture risk assessment (Boutroy et al., 2008). FEA can either be performed using so-called micro FE (μFE) approach or homogenized FE (hFE). While μFE converts each voxel to a hexahedral element, thus leading to high computational costs, hFE makes use of local bone volume fraction (ρ) and anisotropy (fabric) to assess bone properties, leading to comparable values within a significantly reduced computational effort (Pahr and Zysset, 2009). Evidences have shown high correlations between hFE prediction and experimental tests on fresh frozen samples (Varga et al., 2011, Hosseini et al., 2017, Arias-Moreno et al., 2019, Schenk et al., 2022, Simon et al., 2024). Thus, HR-pQCT-based hFE can complement DXA in fracture risk estimations. However, hFE relies on bone quality at the structural level, i.e. fabric–elasticity relationships developed for functionally adapted bone (Zysset et al., 1998). Briefly, fabric–elasticity relationships stands for the relations between the bone morphology and apparent (or continuum) mechanical properties. It was shown that these fabric–elasticity relationships are valid for the trabecular bone in the radius, the vertebra, and the femur (Gross et al., 2012, Panyasantisuk et al., 2015) and hold even in the case of osteogenesis imperfecta (OI) (Simon et al., 2022). Thus, the present study aims to compare the trabecular bone microstructure of non-diabetic and T2D bone samples and test the hypothesis of similar fabric–elasticity relationships. Similar fabric–elasticity relationships will allow to further extend the application of HR-pQCT-based hFE to T2D bone.

## 2 Material and methods

This technical note can be seen as an extension of previously published work on healthy and OI bones (Simon et al., 2022). Thus, most of the methods are similar and will be summarized here.

### 2.1 Participants, samples, and imaging

The control (Ctrl) and diabetic (T2D) groups consisted of 28 donors each. The control group was composed of 14 males and 14 females aged between 51 and 97 years old at death with a mean age of 73 ± 13 years. The diabetic group also counted 14 males and 14 females aged between 54 and 97 years old at death, with a mean age of 75 ± 13 years. The fresh-frozen cadaveric femurs were procured from two tissue banks in the US, Musculoskeletal Tissue Foundation (MTF) and National Disease Research Interchange (NDRI). The accompanied notes for the diabetic donors listed their disease state as type-2 diabetic mellitus (DM) and DM progression for at least 10 years (22 ± 9.9 years). Eight diabetic donors (4 male and 4 female) were also reported to have a stage 3 to 5 chronic kidney disease (CKD). Most of the donors had their DM managed via oral medications such as Metformin, Humalog, and Glipizide. Hypertension and hyperlipidemia also seemed like a prevalent problem across both donor cohorts.

A cylindrical sample of trabecular bone about 10 mm in diameter and 21 mm in height was collected from each donor’s femoral head (left or right). Briefly, the proximal femur was cut to obtain a cube like section that was gripped medially at roughly 25 degree in an angle vise, mounted on a floor drill press, to ensure that the coring axis aligned with the principal orientation of the trabeculae. A diamond embedded trephine drill (TWDCD1150, Eternal Tools, UK) extracted the trabecular core from the posterior quadrant while the bone stayed under constant hydration. The rough core was further trimmed to the exact dimensions by making parallel cuts at either end with a water-irrigated, low-speed circular bone saw. The final cylindrical core was flushed with distilled water to remove any excess marrow and stored in phosphate buffered saline (PBS) at 7.4 pH and −20C until further imaging. The samples were thawed to room temperature prior to imaging. They were loaded into tube holders (Part no. U50813 ø14 mm × 70 mm L) filled with PBS and secured using foam such that the long axis of the core aligned with the scanning axis. The samples were imaged by micro-computed tomography (μCT50, Scanco Medical AG) with the following settings: voltage of 70 kVp, 200 μA, 0.5 mm Al filter, 1 s integration time, and an acquisition rate of 1024 samples per 1000 projections per 360 deg rotation of the tube holder, leading to an isotropic voxel size of 14.8 μm.

### 2.2 Region of interest

In each scanned sample, three cubic regions of interest (ROIs) of 5.3 mm were selected. For this, the image was divided into three stacks of 5.3 mm (top, center, and bottom), and a ROI was selected at the center of mass of the stack.

### 2.3 Morphological analysis

**Fig. 1:**
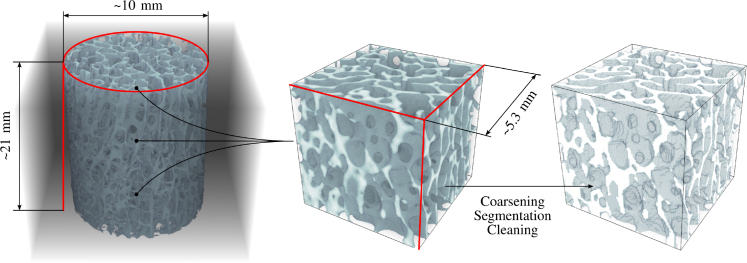
3-dimensional rendering of a typical trabecular core sample, a selected cubic region of interest (ROIs) of 5.3 mm side length and the result of the preprocessing steps. The trabecular core sample and the selected ROI have an isotropic voxel size of 14.8 μm and the coarsened ROI has an isotropic voxel size of 59.2 μm.

Image analysis was performed using medtool (v4.8; Dr. Pahr Ingenieurs e.U., Pfaffstätten, Austria). The pipeline was defined as coarsening, segmentation, cleaning, and morphometry. After selection, the ROI resolution was coarsened by a factor 4 to mimic the 61 μm voxel size available with HR-pQCT. The segmentation was performed using a single threshold based on the average Otsu threshold (Otsu, 1979) of all the scans. The cleaning step consisted of removing isolated islands resulting from the single threshold segmentation. A 3-dimensional rendering of a typical sample, a selected cubic ROI, and the result of preprocessing are shown in Fig. 1.

Then, standard trabecular morphometric parameters were computed. Namely, the bone volume fraction (ρ), trabecular thickness (Tb.Th.), trabecular spacing (Tb.Sp.), and trabecular number (Tb.N.). Additionally, the fabric tensor M was computed using the mean intercept length (MIL) method (Moreno et al., 2014). The ROI’s degree of anisotropy (DA) was computed by dividing the fabric tensor’s highest eigenvalue by the lowest. A last morphological parameter, the coefficient of variation (CV), assessing the homogeneity of mass distribution within the ROI was computed as defined by Panyasantisuk et al. (2015). Briefly, the ROI was divided into 8 cubic sub-volume of identical size and the ρ of each sub-volume was computed. The CV was then computed as the standard deviation of the ρ of the 8 sub-volumes divided by their mean.

### 2.4 Numerical analysis

**Fig. 2:**
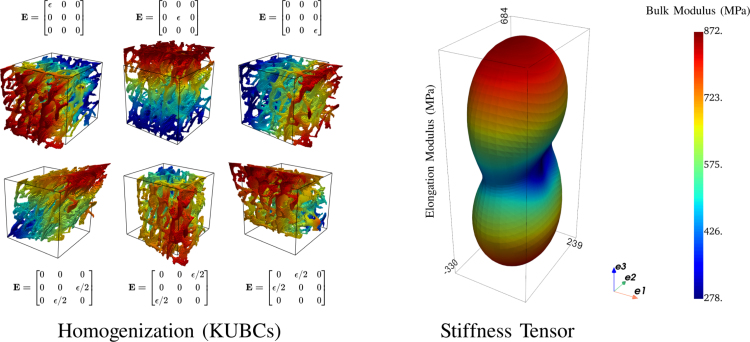
Schematic representation of the homogenization process leading to the ROI’s stiffness tensor. Left, illustration of the 6 loadcases with their corresponding apparent strain tensor. Note that the deformation ϵ should be very small and is amplified by multiple orders of magnitude in the representation. Right, typical stiffness tensor. The shape represents the elongation modulus and the color the bulk modulus (He and Curnier, 1995).

After morphological analysis, each ROI underwent numerical homogenization. For this, μFE analyses were performed using ABAQUS 2023. The model was built using a direct voxel conversion approach to fully integrated linear brick elements (C3D8). Each element was assigned a Young’s modulus of 10 GPa and a Poisson’s ratio of 0.3. These parameters were the same for both healthy and diabetic bone ROIs, thus allowing the comparison of the apparent stiffness due to the architecture only. The homogenization scheme was composed of three uni-axial tests and three pure shear tests using kinematic uniform boundary conditions (KUBCs) (Panyasantisuk et al., 2015). For this, the displacement of each node placed on an external face is prescribed to impose an apparent, homogeneous deformation to the ROI. Then, the ROI’s homogenized stiffness tensor S was computed from these six independent loadcases. This step is illustrated in Fig. 2. Finally, the resulting fully anisotropic stiffness tensor was transformed into the fabric coordinate system and projected onto orthotropy, leading to 12 non-zero symmetric components.

### 2.5 Group comparison

The first comparison between the control and diabetic groups was regarding their morphology. For this, the Mann–Whitney test was performed on samples having a bone volume fraction (ρ) lower than 0.5. Indeed, a ρ higher than 0.5 cannot really be considered as purely trabecular bone. ROIs having a ρ > 0.5 leads to unrealistic morphometric values for trabecular bone and thus, have to be excluded. A p-value lower than 0.05 was considered meaning a significant difference between the groups.

The second comparison was regarding the stiffness tensors after transformation into the fabric coordinate system. The orthotropy assumption was verified by computing the relative norm error between the fully anisotropic tensor and its orthotropic projection. As the assumption of orthotropy is quite common for healthy trabecular bone (e.g. Zysset (2003)), the relative norm error of both group was compared using a t-test to ensure it is also suitable for diabetic bone. After projection onto orthotropy, the individual components of the stiffness tensors were compared. Mann–Whitney test was again used, and a p-value lower than 0.05 was considered significant. These comparisons were performed on samples with a ρ < 0.5 and a coefficient of variation (CV) lower than 0.263. Indeed, this CV threshold was determined by Panyasantisuk et al. (2015) as a limit for the homogeneity assumption. A higher CV involves heterogeneous mass distribution, which violates the representative volume element (RVE) homogeneity assumption (Cowin and Doty, 2007) underlying our calculations.

The third comparison of the two groups was performed by fitting the orthotropic stiffness tensor to the Zysset-Curnier model (Zysset and Curnier, 1995) and comparing the resulting parameters and their 95% confidence interval (95% CI). Briefly, this model expresses the stiffness tensor S based on the bone volume fraction ρ, fabric tensor eigenvalues $m_{1}<m_{2}<m_{3}$, three stiffness constants $\lambda_{0}$, $\lambda_{0}^{\prime }$, and $\mu_{0}$ and two exponents k and l. The fitting procedure consisted of a multiple linear regression which was performed on the logarithmic space as shown in Eq. (1), where $\lambda_{ij}$ and $\mu_{ij}$ are the components of the stiffness tensor, $\lambda^{*}=\lambda_{0}+2\mu_{0}$, and $\delta_{i}$ the residuals. However, as k and l are exponents, it is necessary to impose values for further fabric–elasticity relationship comparison. These imposed values can either be on the exponents (as done in previous work (Simon et al., 2022)) or on the stiffness constants. In the present technical note, it was chosen to impose values for $\lambda_{0}$, $\lambda_{0}^{\prime }$, and $\mu_{0}$, thus assuming identical tissue properties, and compare the resulting k and l. The imposed values were determined by performing the fit on the control and diabetic groups pooled together.

(1) $$\ln\left(\begin{array}{c} \lambda_{11}\\ \lambda_{12}\\ \lambda_{13}\\ \lambda_{21}\\ \lambda_{22}\\ \lambda_{23}\\ \lambda_{31}\\ \lambda_{32}\\ \lambda_{33}\\ \mu_{23}\\ \mu_{31}\\ \mu_{12} \end{array}\right)=\left(\begin{array}{ccccc} 1 & 0 & 0 & \ln\left(\rho \right) & \ln\left(m_{1}^{2}\right)\\ 0 & 1 & 0 & \ln\left(\rho \right) & \ln\left(m_{1}m_{2}\right)\\ 0 & 1 & 0 & \ln\left(\rho \right) & \ln\left(m_{1}m_{3}\right)\\ 0 & 1 & 0 & \ln\left(\rho \right) & \ln\left(m_{2}m_{1}\right)\\ 1 & 0 & 0 & \ln\left(\rho \right) & \ln\left(m_{2}^{2}\right)\\ 0 & 1 & 0 & \ln\left(\rho \right) & \ln\left(m_{2}m_{3}\right)\\ 0 & 1 & 0 & \ln\left(\rho \right) & \ln\left(m_{3}m_{1}\right)\\ 0 & 1 & 0 & \ln\left(\rho \right) & \ln\left(m_{3}m_{2}\right)\\ 1 & 0 & 0 & \ln\left(\rho \right) & \ln\left(m_{3}^{2}\right)\\ 0 & 0 & 1 & \ln\left(\rho \right) & \ln\left(m_{2}m_{3}\right)\\ 0 & 0 & 1 & \ln\left(\rho \right) & \ln\left(m_{3}m_{1}\right)\\ 0 & 0 & 1 & \ln\left(\rho \right) & \ln\left(m_{1}m_{2}\right) \end{array}\right)\left(\begin{array}{c} \ln\left(\lambda^{*}\right)\\ \ln\left(\lambda_{0}^{\prime }\right)\\ \ln\left(\mu_{0}\right)\\ k\\ l \end{array}\right)+\left(\begin{array}{c} \delta_{1}\\ \delta_{2}\\ \delta_{3}\\ \delta_{4}\\ \delta_{5}\\ \delta_{6}\\ \delta_{7}\\ \delta_{8}\\ \delta_{9}\\ \delta_{10}\\ \delta_{11}\\ \delta_{12} \end{array}\right)$$

The fit quality was assessed using the adjusted Pearson correlation coefficient squared ($R_{adj}^{2}$) and relative norm error of fourth-order tensors (NE). This relative norm error allows quantifying the accuracy of the fit. Thus, the multiple linear regression was performed in three different steps.

1. Multiple linear regression with control and diabetic pooled together
2. Multiple linear regression with control group or diabetic group separated allowing fit quality comparison
3. Multiple linear regression with control group or diabetic group separated and imposing $\lambda_{0}$, $\lambda_{0}^{\prime }$, and $\mu_{0}$ from step 1, allowing exponents (k and l) comparison

In order to properly compare the two groups, it is necessary to perform the multiple linear regression on similar ranges of values. In this regard, additionally to filter ROIs according to ρ and CV, a matching was performed between the ROIs of both groups for ρ and degree of anisotropy (DA), where each ROI of the diabetic group was matched to the closest control ROI regarding ρ and DA. To summarize, three subsets were used for the different comparisons:

1. Morphological: ROIs having a ρ < 0.5
2. Mechanical: ROIs have a ρ < 0.5 and CV < 0.263
3. Morphology-mechanical relations: diabetic-control matched ROIs having both a ρ < 0.5 and CV < 0.263

## 3 Results

**Fig. 3:**
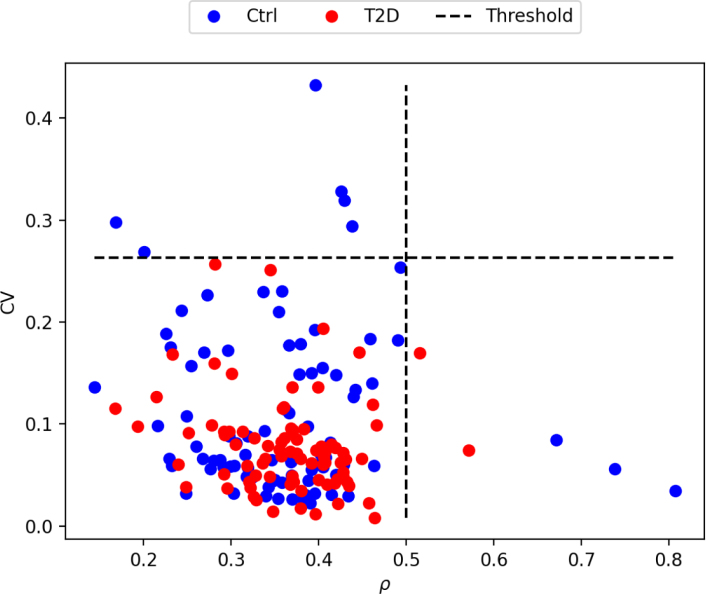
Coefficient of variation (CV) as function of the bone volume fraction (ρ) of the selected regions of interest (ROIs). The dashed black lines show the threshold used to filter ROIs that do not meet the model assumptions.

Fig. 3 shows the distribution of the ROIs selected according to the bone volume fraction (ρ) and coefficient of variation (CV). Filtering ROIs with a bone volume fraction that is too high leads to the exclusion of two diabetic and three control ROIs. The further filtering according to CV for mechanical and morphology-mechanical relations comparison leads to the additional exclusion of two diabetic and four control ROIs. Finally, matching ROIs for ρ and DA leads to 76 ROIs in each group.

### 3.1 Morphology and mechanics

Morphological comparisons are shown in Table 1. For each of the analyzed trabecular parameters, the interquartile range (0.25–0.75) presents a significant overlap between the control and diabetic groups. This overlap is confirmed by the p-value resulting from the Mann–Whitney test being higher than the significant level of 0.05 for all computed variables. The relative norm error of orthotropy and the component-wise comparisons of the stiffness tensors resulting from homogenization are available in Appendix. As for the morphological comparison, none of the variables compared (orthotropy assumption relative norm error and stiffness components) shows a significant difference (p-value always higher than 0.05) between the control and the diabetic group.

**Table 1:** Summary of the morphological parameters and comparison. The first column shows the variable assessed, the second column is the p-value resulting from the Mann–Whitney test, and the third and fourth columns show the median value for each group with their interquartile range.

Variable	p-value	Ctrl	T2D
ρ (–)	0.77	0.36 [0.31–0.41]	0.37 [0.30–0.40]
Tb.N. (1/mm)	0.06	1.02 [0.93–1.06]	1.04 [0.96–1.12]
Tb.Th. (mm)	0.41	0.31 [0.28–0.33]	0.30 [0.28–0.32]
Tb.Sp. (mm)	0.09	0.68 [0.63–0.75]	0.66 [0.59–0.74]
Tb.Sp.SD (mm)	0.50	0.07 [0.07–0.09]	0.07 [0.07–0.08]
DA (–)	0.29	1.66 [1.54–1.81]	1.69 [1.59–1.82]
CV (–)	0.94	0.07 [0.05–0.14]	0.07 [0.05–0.11]

### 3.2 Morphology-mechanical relationships

The multiple linear regression fitting control and diabetic group pooled together to the Zysset-Curnier model lead to an $R_{adj}^{2}$ of 0.97 and a norm error of 0.08. The parameters $\lambda_{0}$, $\lambda_{0}^{\prime }$, and $\mu_{0}$ were 3959, 3253, and 3413 MPa, respectively. The fits performed on the individual groups are shown in Fig. 4. The control group reaches an $R_{adj}^{2}$ of 0.97 and a norm error of 0.08, as for the regression using the two groups pooled together. The multiple linear regression performed on the samples from diabetic patients alone reaches a slightly higher $R_{adj}^{2}$ of 0.98 and a norm error of 0.08.Fig. 4Results of the multiple linear regression performed on the individual groups. Observed S is the stiffness tensor resulting from numerical homogenization, and fitted S is the stiffness tensor predicted by the theoretical model.

**Fig. 4(a):**
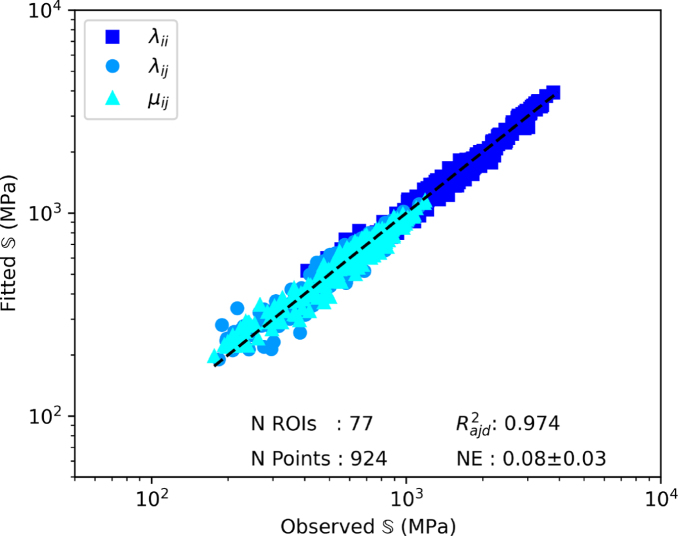
(a) Control group.

**Fig. 4(b):**
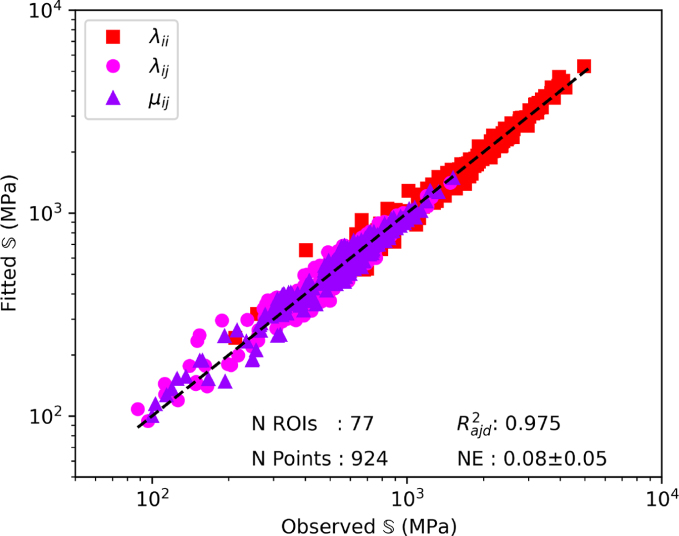
(b) Diabetic group.

**Fig. 5:**
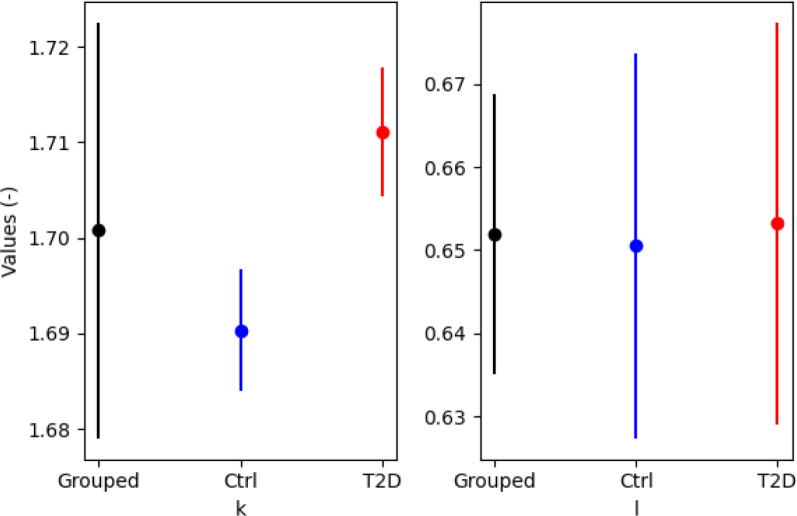
The resulting exponents and their 95% confidence interval of the restricted multiple linear regression performed on the grouped and individual groups.

**Fig. 6:**
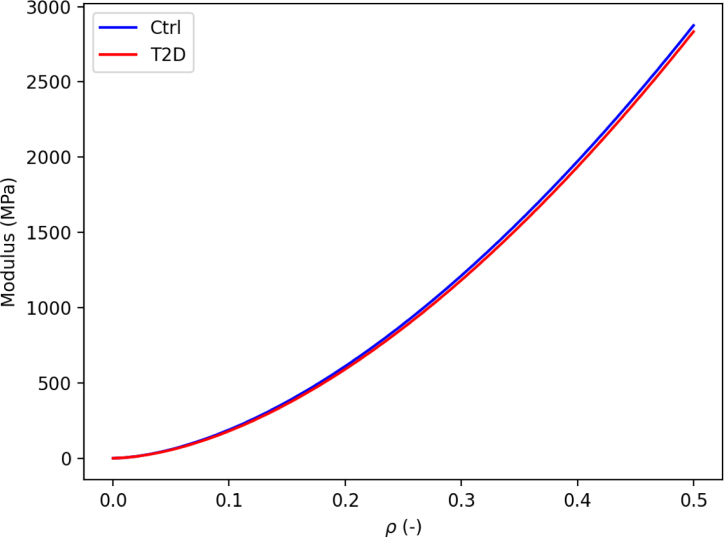
Evolution of Young’s modulus as function of bone volume fraction ρ according to the fabric–elasticity model using the different k exponents obtained by linear regression for the control and diabetic group.

Finally, a comparison of the values obtained for the k and l exponents for restricted multiple linear regressions are shown in Fig. 5. Pooling control and diabetic group together lead to k and l values of 1.7 (1.68–1.72) and 0.65 (0.64–0.67) (value and 95% CI), respectively. The separation of the groups leads to lower k value for the control group than for the diabetic group, and their 95% CI do not overlap but the l exponent presents well overlapping 95% CI for both group. The control versus the diabetic group shows a k of 1.69 (1.68–1.70) versus 1.71 (1.70–1.72) and an l of 0.65 (0.63–0.67) versus 0.65 (0.63–0.68), respectively. While being different, the relative difference in k exponent between the two group is about 1%. The effect of this difference on the theoretical Young’s modulus based on the fabric–elasticity model as function of the bone volume fraction is shown in Fig. 6.

## 4 Discussion and conclusion

This technical note investigates relationships between morphology and elasticity of trabecular bone in diabetic conditions as compared to control. This work can be seen as an extension of previous similar work (Simon et al., 2022) comparing osteogenesis imperfecta conditions to healthy controls. Briefly, the trabecular morphology of regions of interest (ROIs) from femoral head samples is analyzed. Then, numerical homogenization using identical material properties allows comparing the apparent stiffness due to the architecture of these ROIs. Finally, the fitting to a fabric-based orthotropic model (Zysset and Curnier, 1995) allows comparison of fabric–elasticity, i.e. morphology-mechanical relationships between samples from the control or the diabetic group.

To be considered as trabecular bone regions, ROIs presenting a bone volume fraction (ρ) higher than 0.5 were excluded from the analysis. The presence of cortical bone within these excluded ROIs might come from the original anatomical location of the samples and, to a reduced extent, the choice of the single threshold value for segmentation. The comparison of morphological parameters between the control and diabetic groups shows no significant difference. These results mostly agree with similar morphological comparisons performed using HR-pQCT (Burghardt et al., 2010, Shu et al., 2012, Farr et al., 2014, Paccou et al., 2015, Samelson et al., 2018, van Hulten et al., 2024). While some of these studies have shown no differences between control and T2D (Shu et al., 2012, Samelson et al., 2018, van Hulten et al., 2024), the significant p-values were comprised between 0.05 and 0.01 in the studies of Burghardt et al. (2010) and Paccou et al. (2015) and significant differences vanished after adjustment for body mass index (BMI) in the study of Farr et al. (2014). In the present study, specifically ρ, Tb. Th., Tb. Sp. SD, DA and CV are highly unlikely to be different. Thus, the morphology of femoral head trabecular bone samples presented in this work is highly similar in diabetic conditions as compared to control.

Homogenization of the cubic ROIs requires that the bone mass is homogeneously distributed with the volume. Therefore, ROIs presenting a CV higher than a previously defined threshold of 0.263 (Panyasantisuk et al., 2015) were filtered out. The component-wise comparison of the resulting stiffness tensors shows no differences between ROIs from control or diabetic patients. This result complements the morphological comparison, showing that, beyond similar morphology, the mechanical behavior resulting from the bone structural quality is similar between diabetic and control samples.

After morphological and mechanical comparison, the relationships between morphology and mechanics are compared using the Zysset-Curnier model (Zysset and Curnier, 1995). As performed in previous work (Simon et al., 2022), ROIs are matched for ρ and DA to perform the fit on similar ranges for both groups. The fit quality assessed using $R_{adj}^{2}$ and NE is in the expected range, i.e. similar as observed in other studies (Gross et al., 2012, Panyasantisuk et al., 2015, Simon et al., 2022) Moreover, these quality parameters are quite similar among the different data sets used for the regression (grouped, control, and diabetic), representing a first step indicating similar fabric–elasticity relationships between control and diabetic conditions. Going one step further, stiffness parameters ($\lambda_{0}$, $\lambda_{0}^{\prime }$, and $\mu_{0}$) are fixed to a common value, assuming identical bulk tissue properties, and the resulting exponents (k and k) with their 95% CI are compared. The k exponent tend to be higher for the diabetic group than the control group, and their 95% CI do not overlap but the 95% CI of the l exponents shows important overlap. Thus, the l value, which is directly linked to the fabric, could be assumed to be the same for both groups. On the other hand, the 95% CI for the k exponent, which is linked to the bone volume fraction ρ, is different between the groups. Indeed, it is worth noting that the k exponent tends to be closer to 1 for control group than for the diabetic group, indicating a more efficient organization of the bone mass. However, this difference is relatively small, leading to extremely similar curves of Young’s modulus as function of the bone volume fraction. Altogether, these results allow assuming that fabric–elasticity relationships are similar between non-diabetic and diabetes-diagnosed individuals.

Nevertheless, this study presents some limitations that need to be addressed. First, the samples were harvested only from the femoral head, thus limiting the generalization of the results to other anatomical locations. However, multiples studies have shown that the fabric–elasticity relationships are extremely robust and hold similarly across anatomical locations (Gross et al., 2012, Panyasantisuk et al., 2015, Simon et al., 2022). Second limitations are the downsampling of the original ROIs and the filtering steps. Indeed, the downsampling was performed to mimic the resolution of HR-pQCT, which is the clinical tool used for bone health assessment. This downsampling lead to bulkier structures, which might have slightly modified the morphological and mechanical properties. However, this downsampling was performed on both the control and diabetic ROIs similarly, thus not affecting the comparison between the two groups. Regarding the filtering steps, the exclusion of ROIs with a bone volume fraction higher than 0.5 is necessary to ensure that the ROIs are purely trabecular bone. Additionally, the filtering step removing highly heterogeneous ROIs is also a necessary step because it ensures that the ROIs being analyzed are statistically representative of trabecular bone. Third, this work is limited to numerical simulations only and does not include experimental validation. However, validation of this approach was done by Rincon-Kohli and Zysset (2009) and the main focus of this work is the comparison of fabric–elasticity relationships between healthy and diabetic individuals, for which numerical simulations provide a robust comparison. Finally, the medical history of the two cohorts is limited. While the diabetes duration and the medication of the T2D group is known, no detailed information is available about the potential diseases of the control group. However, these missing information are not thought to have a significant impact on the results of the present study as the objective is compare diabetic to non-diabetic conditions.

To conclude, the present work shows that trabecular bone from the femoral head of diabetic conditions presents similar morphology, mechanics, and fabric–elasticity relationships compared to control. As mentioned in the introduction, these fabric–elasticity relationships are a basic assumption of HR-pQCT-based hFE simulations. Therefore, the present results suggest using HR-pQCT for bone health assessment and monitoring in diabetes-diagnosed patients.

## CRediT authorship contribution statement

**Mathieu Simon:** Writing – original draft, Validation, Software, Methodology, Investigation, Formal analysis, Data curation. **Sasidhar Uppuganti:** Writing – review & editing, Resources, Data curation. **Jeffry S. Nyman:** Writing – review & editing, Validation, Resources, Project administration, Funding acquisition, Conceptualization. **Philippe Zysset:** Writing – review & editing, Validation, Supervision, Resources, Project administration, Methodology, Funding acquisition, Conceptualization.

## Research ethics

We further confirm that any aspect of the work covered in this manuscript that has involved human patients has been conducted with the ethical approval of all relevant bodies and that such approvals are acknowledged within the manuscript.

## Funding

This work was funded by the 10.13039/501100001711Swiss National Science Foundation, Switzerland (SNSF), grant number 200365, Department of Veterans Affairs, Biomedical Laboratory Research and Development Service, grant number BX004297, and the 10.13039/100000069National Institute of Arthritis and Musculoskeletal and Skin Diseases, United States , grant number AR063157.

## Declaration of competing interest

The authors declare the following financial interests/personal relationships which may be considered as potential competing interests: Philippe Zysset reports financial support was provided by Swiss National Science Foundation (SNSF). Jeffry S Nyman reports financial support was provided by Department of Veterans Affairs, Biomedical Laboratory Research and Development Service. Jeffry S Nyman reports financial support was provided by National Institute of Arthritis and Musculoskeletal and Skin Diseases. If there are other authors, they declare that they have no known competing financial interests or personal relationships that could have appeared to influence the work reported in this paper.

## Figures and Tables

**Fig. 7:**
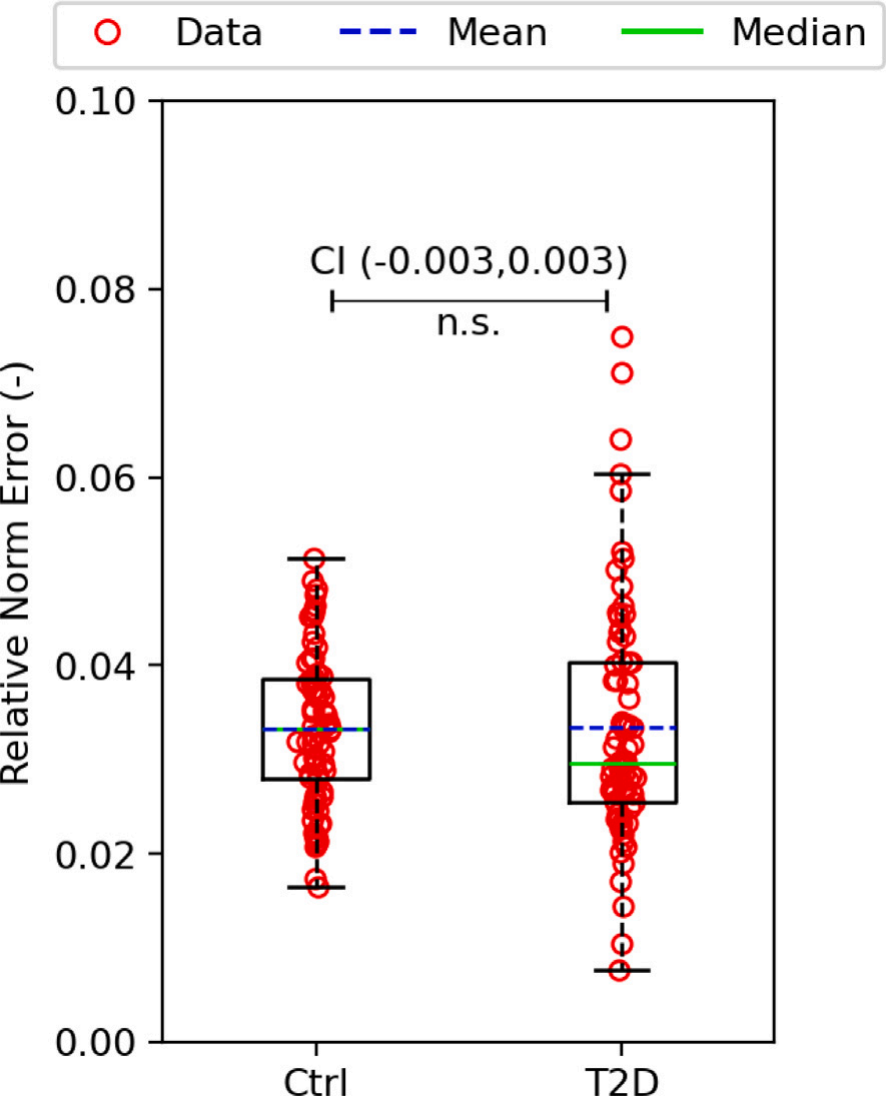
Relative norm error between the fully anisotropic stiffness tensor and its orthotropic projection. CI is the 95% confidence interval of the t-test and n.s. stands for non-significant p-value.

**Figure:**
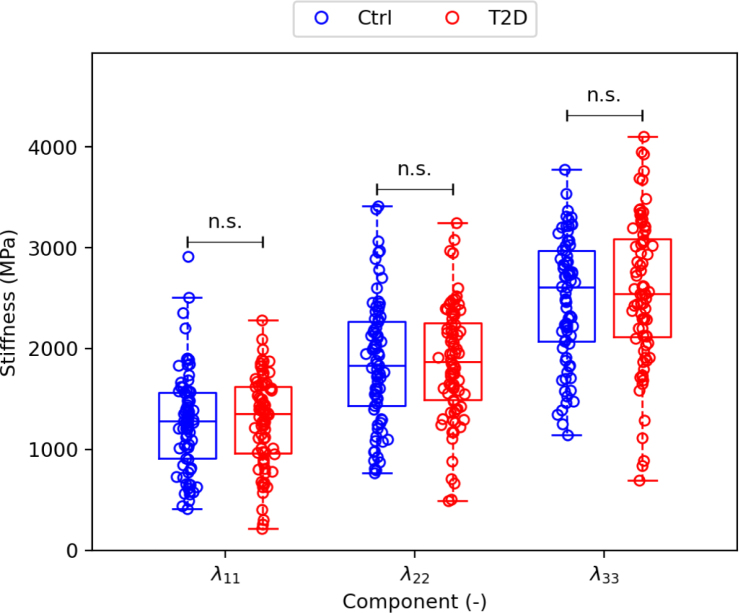


**Figure:**
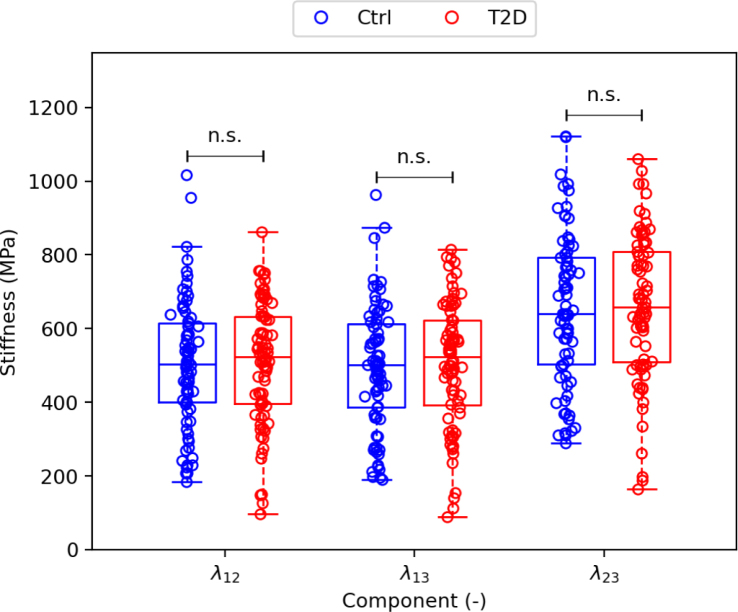


**Figure:**
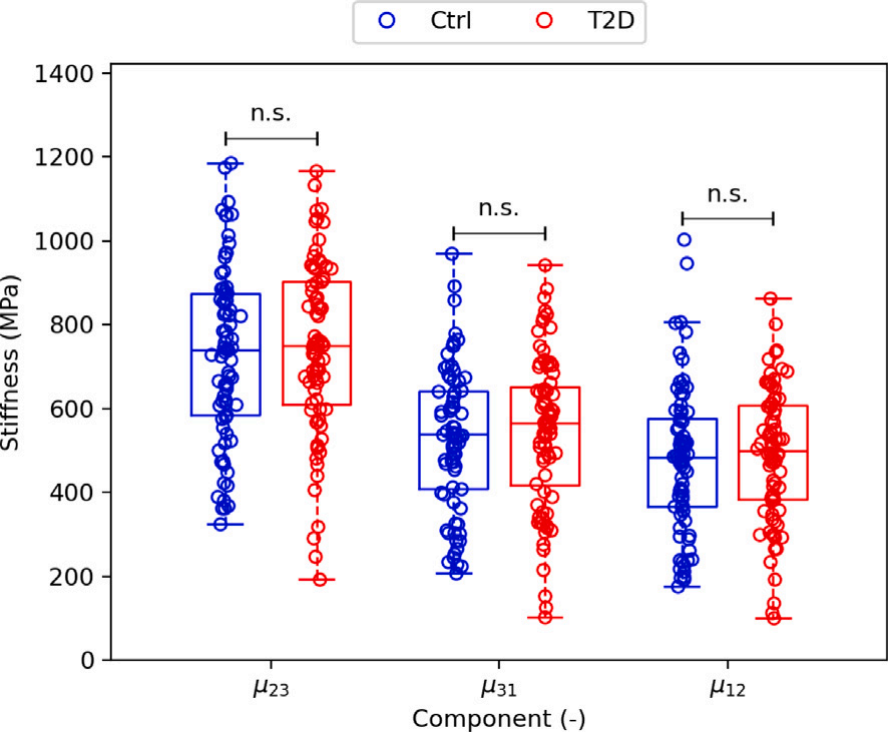


## Data Availability

The data that support the findings of this study are available on request. The data are not publicly available due to privacy/ethical restrictions. The scripts used for the analyses performed in the present study are available on GitHub: https://github.com/artorg-unibe-ch/FABTIB.

## Appendix Mechanical comparison

The results of the orthotropy relative norm error and the component-wise comparison for each non-zero component of the apparent stiffness tensor resulting from homogenization are shown in Fig. 7, Fig. 8.
